# “It Is Scary, but Then I Just Do It Anyway”: Children’s Experiences and Concerns about Risk and Challenge during Loose Parts Play

**DOI:** 10.3390/ijerph20227032

**Published:** 2023-11-07

**Authors:** Martin van Rooijen, Kristine De Martelaer, Gerty Lensvelt-Mulders, Lisette van der Poel, Mieke Cotterink

**Affiliations:** 1 Department of Education, University of Humanistic Studies, 3512 HD Utrecht, The Netherlands; 2 Department of Movement & Sport Sciences, Vrije Universiteit Brussel, 1050 Brussel, Belgium; kdmartel@vub.be; 3 Department of Research Methodology and Theory of Sciences, University of Humanistic Studies, 3512 HD Utrecht, The Netherlands; g.lensvelt-mulders@uvh.nl; 4 Research Group Youth, University of Applied Sciences, 3584 CH Utrecht, The Netherlands; lisette.vanderpoel@hu.nl; 5 Consumer Safety Institute, 1062 XD Amsterdam, The Netherlands; m.cotterink@veiligheid.nl

**Keywords:** children’s voices, risky play, elementary school children, intervention program, qualitative research, loose parts play, self-determination theory, outdoor play, unstructured play

## Abstract

Children’s risky play opportunities depend on supervising adults’ attitudes and the play environment. The possibilities to engage in risk-taking outdoor play for children have seriously decreased over the last few decades, due to safety concerns and adults’ preoccupation with protection. In response to this shift, research has increasingly focused on influencing factors on professional attitudes toward risk-taking in children’s play. However, children’s perspective on risky play is underrepresented in the recent literature. This study generates awareness of children’s risky play preferences and interests to help professional caretakers hone their facilitating role. We explored children’s notions of risk and challenge in play during a loose parts intervention stimulating risky play and facilitated by after-school childcare practitioners. A thematic analysis examined observations, informal conversations, and roundtable talks with children about their risky play experiences. Children describe their risk-taking in play as experimental and daring. The findings report on children’s general views on risky play, their play experiences with loose parts, their real-life risky play experiences, and their opinions on the role of practitioners. By relating the results to risky play research and self-determination theory, this study offers insight into children’s innate needs. Taking risks on their own terms gives children a sense of self-confidence and mastery, and forces them into new relationships with other children and guiding adults. Consequently, children fulfill the three universal needs of self-determination theory: autonomy, competence, and relatedness.

## 1. Introduction

Risk is a major concept in modern Western society and is predominantly perceived as something negative to be avoided, leading to hazard-based approaches to everyday situations [1,2,3,4]. This attitude toward risk has consequences for the way children are approached in educational and nurturing settings. Children are perceived as vulnerable and prone to accidents, leading to safety concerns and adults’ preoccupation with protection [5,6]. These perceptions have been implicated in the declining opportunities for children to engage in risky play. Recent research has shown that children’s possibilities to independently engage in challenging and risk-taking outdoor play have seriously decreased over the last few decades [6,7,8,9]. Adults who can recall experiences of ultimate freedom to play in their own childhoods find it difficult to give their own children the same room for exploration [10]. In this context, research shows that if children are free to select the level of risk in their play activities, they will frequently choose a higher level than the guiding adult would predict and consider acceptable [11]. A lack of opportunities for risky and challenging play has negative consequences for becoming a healthy adult, such as learning to trust oneself, recognizing one’s limits, and knowing when it is better to ask for support [12]. 

In response to this shift, research has increasingly focused on children’s risk-taking and motivation for engaging in risky play [7,9,13]. Recent discourse has raised questions about the approach toward risky play, about who defines risk, and about how adults engage with children and discuss with them their risk competencies and understanding of risky practice. It has been argued that children’s play has become subject to adult scrutiny and is no longer something children just do, with adults controlling children’s play and removing children’s agency to determine their own play [5]. Supervising adults habitually rush judgments on risky play, which has a negative impact by inhibiting children’s challenging play activities [14]. In general, children have a relatively boundless view of their playing opportunities, but they frequently say that adults restrict their play possibilities [15]. Glenn et al. argue that adults should facilitate rather than hinder children’s play by providing children with choice and agency and by allowing them to retain the spontaneity associated with outdoor play. 

The scope of this paper is elementary-school-aged children’s understandings of risky play. Related contemporary research has examined children’s own understanding of their well-being in childcare settings [16,17,18]. This child-centric study is in line with the increasing attention given to children’s own views in research about their life-worlds [19,20,21], specifically their own ideas on risky play [22]. 

This paper examines children’s perceptions of risky play and describes the outcomes of their experiences in a professional environment (i.e., after-school childcare), where loose parts were introduced to provide additional opportunities for risky play. The goal of this study is twofold: firstly, to contribute to existing theories on risky play and self-determination; and secondly, to achieve an applied goal with societal relevance by supporting professional caretakers in facilitating risky play. In this article, we use the term *practitioner* as an all-encompassing term for professional and voluntary supervisors of children in staffed environments, such as childcare or after-school activities.

## 2. Theoretical Background

### 2.1. Developmental Values of Risky Play in Childhood

Firstly, it is important to understand the developmental values of risky play in childhood. Sandseter’s definition of risky play has been widely accepted in international research as a “*thrilling and exciting form of play that involves a risk of physical injury*” [23]. Importantly, the definition refers to physical risks, not social perils. Children show a common preference for risky play when choosing between typical play types; girls and boys equally practice risky play both outdoors and indoors [6]. Notably, a wide range of such risk experiences is important for children’s well-being in many aspects: “*It helps them keep healthy and enhances their resilience, enables them to develop and learn, influences their perception of themselves and their self-esteem, and provides excitement and pleasure*” [24]. In this context, Cooke et al. [25] describe beneficial risk as engaging in experiences that take a person out of their comfort zone and include outcomes that may be beneficial to learning, development, and life satisfaction. Play containing uncertainty allows children to position themselves in situations that convey a feeling of risk without overexposing them to the serious possibility of injury [26,27]. When they create risky play situations, children are in control while experiencing the sensation of being out of control. Therefore, children need space, both socially and physically, to be active and engage in challenges. It is necessary that they take risks in their play to develop “risk competence,” which refers to “*the process of becoming knowledgeable and skilled in assessing risks and therefore acquiring the competence to take risks more safely*” [24]. The educational foundation for building risk competence is that children can protect themselves and make the right choices on their own. Malaguzzi [28] mentions the concept of the “rich child,” whereby children are seen as competent and resourceful with a richness of skills, knowledge, and capabilities. Moreover, eradicating risk in play is literally impossible; likewise, it withholds from children essential experiences to develop resilience by experimenting, exploring their capabilities, and mastering new activities [29]. By limiting opportunities for risk-taking, adults are also depriving children of opportunities to strengthen their resilience and their ability to cope with stress and uncertainty. This can lead to anxiety and other mental health issues and may cause children to avoid new experiences [30,31]. 

### 2.2. Self-Determination Theory and Risky Play

In addition to understanding the developmental values of risky play in childhood, this theoretical framework applies self-determination theory to risky play. Self-determination theory (SDT), developed by Deci and Ryan [32,33], proposes a framework for how to better understand and promote children’s optimal development and autonomy [34]. SDT has previously been applied to children’s play, exploring adult influences on children’s perceptions of choice when they play [35]. With both a biological and psychological perspective, SDT emphasizes important and natural developmental tendencies that can be related to the functioning of children—specifically, risk-taking play. Three indispensable aspects of risky play can be distinguished that are likewise existent in SDT as the roots of motivation. These three basic psychological needs are autonomy, competence, and relatedness (desire to feel connected to others) [32,33]. Firstly, risky play is related to improving children’s autonomy, developing decision-making skills [36], improving agency [37], and enhancing responsibility [38]. Secondly, risk-taking play has been proven to benefit children’s competencies like risk perception, mastering risk, motor control, and courage development [13,39,40]. Thirdly, the possibility of engaging in risky play depends on which interrelated role supervising adults adopt toward children, how much confidence adults project toward the child, and how the child experiences this confidence [41,42]. Moreover, the nature of risky play often involves relating to other children and socially interacting as peers when they engage in challenging activities.

### 2.3. The Role of Practitioners in Supporting Autonomy

After-school childcare can offer optimal opportunities for children to become acquainted with risky play; however, there are protectionist barriers to lift for enabling practitioners to support children in daily practice. A growing amount of children’s time is spent in structured environments, such as school, childcare, or other institutes, where professionals supervise them [17,43,44]. However, the primary responsibility for children lies with their parents. The allocation of duties between parents and professional caregivers (e.g., childcare professionals and teachers) is challenging. Parents expect practitioners mainly to offer protection and to ensure a safe social and physical environment, contrasting with the professional duty of care, which also encompasses responsibility for healthy growing up and thus providing risky play possibilities for children [45,46]. Childcare work is regulated by protocols and controlled by public health services and educational inspections, where health and safety are paramount. 

As indicated above, an emphasis on risk as something to be avoided at all costs does not improve children’s opportunities for challenge and freedom of play and thus limits their opportunities for healthy physical and psychological development [13]. Within the societal discussion about challenges and security for children, there is an argument that a risk–benefit assessment should be linked to pedagogical perceptions [47]. This view originates from a vision that allows children the freedom to grow up in a challenging and development-oriented environment. Practitioners focusing on the adaptability of choice should assist and guide children through play by varying the amount of motivation and beneficial risk, and by doing so, work to increase a child’s actual and potential level [48]. In this view, practitioners should provide children with opportunities to deal with risks and challenges in their play, thus conceding to the natural urge of children to overcome their fear and explore their boundaries in physical play [49]. Indeed, there is a movement for professionals in education and childcare to reconsider their role as risk-avoiders and to prioritize the curiosity and understanding of children over adult expertise [50]. However, factors that can hinder professional attitudes toward risk-taking in children’s play involve practitioners’ own character and their relationship with parents [51]. Likewise, professional attitude, motivation, and actions that help them provide a challenging play environment for children are influenced by caregivers’ understanding of children as well as regulatory and cultural factors [51]. 

Cooke et al. [25] suggest that it is important to help practitioners plan and support opportunities for children’s risk-taking and to increase practitioners’ confidence to develop innovative risky play practices. Consequently, the understanding of risk-associated practices and the development of managing risks during play in childhood must be a focus in daily practice and in higher education for professionals working with children—in particular, in childcare. In line with the notion that beneficial risk-taking in play can support physical, cognitive, social, and emotional abilities [52], curriculum documents and government policy increasingly encourage professionals in childcare institutions to allow children to be risk-takers [53]. In the Netherlands, for example, this policy is phrased as follows: “*We teach children to deal with small risks but protect them against big risks*” [54].

### 2.4. Loose Parts as Affordance for Risky Play

For after-school childcare settings, so-called loose parts are relatively easy to implement by the team, at low-cost, and effective in stimulating children’s risky play. In this study, therefore, a loose parts play (LPP) intervention was introduced. Loose parts are open-ended materials and equipment without well-defined uses. Such parts facilitate unstructured, child-led play. LPP is a technique evolved from playwork practice that makes use of “stuff” like old crates, tires, office chairs, and cable reels in play spaces, inviting children to engage as they prefer with limited adult involvement [55,56]. Loose parts afford maximum opportunities for engagement, and LPP is rooted in the loose parts theory of Nicholson and the theory of affordances of Gibson [57,58,59]. Both theories assume that the number and kind of variables in an environment are directly related to the various opportunities for action or use—and that the affordances (uses) are different for each individual [60]. This philosophical approach attaches the features of outdoor play possibilities (e.g., loose parts) to the bodily and mental propensities of the child [61]. LPP interventions offer extended opportunities for risk-taking, which has positive developmental benefits for children’s competence, social skills, and physical activity [39,62,63]. Like previous research in this study, LPP was chosen to enhance opportunities for risky and challenging play [62,64]. Hence, we provided recycled scrap materials to cater to categories of risky play, such as great heights, rough-and-tumble play, high speed, and disappearing [40]. Items included crates, cable reels, office chairs, ladders, buggies, and tree trunks. 

## 3. Context of this Study, Aim, and Research Questions

In the present study, children’s own constructions and experiences of risk-taking were explored. After-school childcare (also called “out-of-school care” or “school-aged childcare”) is a significant social and play environment for an increasing number of children. In the first quarter of 2020, more than 7400 after-school care facilities existed in the Netherlands, with 409,000 children aged 4 to 12 years attending, or 29% of the primary school population [65]. 

This study adopted a qualitative approach and is part of a larger, mixed-methods intervention study on children’s possibilities of risk-taking play in after-school childcare in the Netherlands and the professional attitudes of the staff supervising this kind of play [66]. The impact of a professionalization development program on facilitating risky play has been described in detail elsewhere [66]. The conclusion of that study was that moral tension existed in the domains of “safety and autonomy” when working with children and in the domains of “unity and diversity” in collaboration with colleagues. Practitioners observed more joint play, play with new play friends, and more communication between children in their risk-taking play with loose parts [66]. For the present study, qualitative data were collected to explore the perceptions of children regarding the intervention. This paper thus expands on the outcomes of risky play for practitioners by focusing on how children experience the modification of their play environment and their perspectives on adult interference in their risk-taking play activities. This study adopts an exploratory approach to assess how children interpret their experiences and evaluates the benefits children realize in their risk-taking play, focusing exclusively on their perspectives. A phenomenological approach was employed, using informal conversations, observations, and roundtable talks to capture children’s lived experiences [67]. Phenomenology appraises descriptions and reflections on practice as essential to understand the nuance and nature of the experience [68]. By applying this avenue in this study, the risky play practices of children were leading and thus, we did not place an emphasis on age, gender, or other contextual factors. Following Smith [69] in his ”pedagogy of risk”, this study does not pursue a representative group of children selected by age or gender, it is their individual perceptions of risk that are of importance in determining the nature of risk-taking responses. In rigorous thematic analysis, the authors engaged in reflective discussion to reveal these experiences in main themes.

The focus of this study was on gaining an understanding of school-aged children’s involvement in a loose parts intervention intended to stimulate risky play and facilitated by childcare practitioners [70,71]. The first aim is to contribute to the literature around risky play and SDT by generating awareness about children’s views about their risky play preferences and interests. The second aim is to explore tools for practitioners to consider regarding their relatedness with children in facilitating more risky play connected to SDT needs. Four research questions were formulated. The first two questions explore the definitions and categories of risky play [40,72] and the theory of loose parts: (1) What do children see as risk-taking in play? (2) What kind of play do children experience using loose parts? The third and fourth questions are grounded in SDT: (3) What experiences do children have with risk-taking during this study? (4) What is the opinion of children about the role of practitioners during risky play? 

In the remainder of this paper, we firstly present the design of this study, including how the data were collected as well as the methodological approach. Secondly, the outcomes of the empirical study are presented in the context of children’s play spaces. Subsequently, it is argued that children’s voices are important for practitioners who are guiding them in their play experiences. 

## 4. Materials and Methods

This study in after-school childcare settings was conducted in the Netherlands from February to June 2018. The research team explored children’s notions of risk and challenge in their play. In this chapter, we describe research participants, data collection methods, and data analysis, and we elaborate on the ethical issues relating to research in children’s space and time. 

### 4.1. Loose Parts Materials

All settings involved were given access to a sea container or shed containing different loose parts for the duration of six weeks. The practitioner team decided when children had access to these parts, varying from one to five afternoons a week, with a duration of two to four hours per play session. Loose parts were collected in collaboration with local recycle shops, which may have led to differences in materials between the locations. However, every storage contained at least the following scraps, thus enabling five of the six categories of risky play [40]:

1. Play with great heights: crates, ladders, stools cable reels, tree trunks;
2. Play with high speed: office chairs, tires, mattresses, buggies, walkers;
3. Play with dangerous tools: ropes, sticks, planks;
4. Rough-and-tumble play: buckets and stretchers, cushions;
5. Out-of-sight play: carpets, garments for making huts.

The category “play near dangerous elements like water and fire” was not included in this study’s facilitation of risky play. Such play cannot be facilitated with loose parts and must be closely supervised. However, children can have experiences with these elements in other contexts, like home.

### 4.2. Participants

Ten childcare organizations responded to a call in a professional national childcare journal. In a later stage, three withdrew for time-investment reasons. The seven remaining childcare institutions, located in different regions of the Netherlands, were selected. The settings varied in size and context (See Table 1).

### 4.3. Data Collection

This study was conducted with the assistance of nine undergraduate student researchers from the bachelor’s pedagogy program (educational theory) at the University of Applied Sciences, Utrecht, under the supervision of the first author. The students could apply for participation in this project to carry out research tasks as part of their curriculum. Prior to the beginning of the research, the students attended a meeting where they were informed by the first author of practical matters, research design, and procedures, including their specific tasks. On an individual basis and in monthly sessions as a group, students were coached by their lecturer (fourth author of this article) in a setting where they could share their experiences. The principal researcher was present at two of these group sessions to teach theories on risky play and visited each research setting monthly to facilitate the student researchers. In addition to a range of possible methods that students were already familiar with, they learned the theory and practice of appropriate research tools to elicit children’s views. The “reactive method” of Corsaro [73] and the “neutral intermediary” approach from Meire et al. [74] were influential in how student researchers were informed. Each student was assigned to an after-school childcare location; two settings received two students for different age groups. The student researchers were present two or three afternoons a week during a period of four months. The first month allowed the student researchers to get to know the children and context; thereafter, they participated in the professionalization program of the team and performed their research tasks before, during, and after the LPP intervention.

To understand children’s risky play experiences, qualitative and interpretivist approaches [75] were used. Data were gathered in multiple ways. Diaries were used for capturing observations and informal conversations with children about their outdoor play behavior. In each setting, a single roundtable talk about their risky play possibilities was conducted with the children.

### 4.4. Procedure

Student researchers were sensitive to children’s risk-taking play, making use of Sandseter’s [72] categorization of risky play and the risky behavior categorization regarding children’s motivation and skills from Little and Eager [76]. The student researchers made notes of their observations and informal conversations as “thick descriptions” in digital diaries [75]. In all settings, in total, 321 notes were taken on children’s play activities. Moreover, at each location, student researchers organized a roundtable talk with four to six children selected by the practitioner team, using a semi-structured topic list [77,78]. As moderators, they were instructed not to explicitly refer to risky play to enable children to use their own vocabulary. The structure of the group conversation, which lasted 15–30 min, consisted of five themes: (1) exciting and challenging play experiences; (2) reasons for and feelings about this play; (3) possibilities for this play at the setting; (4) what practitioners do and say during this play; and (5) differences with the home setting. The interviews with children consisted of often fragmented, ambiguous, and sometimes inconsistent narratives, which echoed the complex reality of children and the way they talk [8,79]. The roundtable talks, with a total of 49 participants aged from 4 to 10 years, 32 males and 17 females, were recorded and transcribed. 

### 4.5. Data Analysis

A thematic analysis was then undertaken, encompassing the reading and re-reading of writings to recognize common themes [80]. Firstly, all primary data were read and re-read thoroughly by the first author (close reading). The quantity and extent of the data varied considerably, depending on the location, the student researcher, and the after-school organization. Therefore, it was decided to draw up the material as a single document comprising all roundtable transcripts, observation notes, and informal conversations. A content analysis was performed on the transcripts to scan the children’s responses to the four research questions to organize the data. Because the present study focuses on exploring children’s experiences with risky play, only elements concerning actual practices were analyzed. This resulted in including 8 transcripts of roundtable talks, 7 observations, and 9 informal conversations before the intervention, as well as 39 observations and 20 informal conversations during the intervention for analysis. All authors, except the second author, then carried out a qualitative thematic analysis to inductively develop a list of codes that were used [81,82,83,84] in consecutive steps. Firstly, close reading was carried out with the goal of becoming familiar with the raw data. Secondly, the researchers coded the text, thus “*reducing the data into meaningful segments and assigning names for the segments*” [84]. In this stage, each of the researchers focused on one of the research questions. Thirdly, axial coding was applied to define subcategories [81]. In the fourth step, emerging patterns were derived through group discussion, reflecting the current research questions, and comparing the most common codes and relations between codes to develop the main themes. Key findings were then discussed between researchers, resulting in full agreement. Those findings are presented in Chapter 5.

### 4.6. Ethical Considerations

This study followed the codes of conduct for academic practice published by the Association of Universities in the Netherlands. The data were stored safely according to the data management policies of the University of Humanistic Studies. Applicable procedures for research in educational settings were used during the period of data collection. Specifically, the following measures were taken to guarantee an ethically responsible research approach. The participating childcare organizations were informed about the loose parts intervention before they agreed to participate. Both organizations and parents received information about the goal of improving children’s risk competence and increasing practitioners’ competence in supervising risky play. Parents were asked for consent that their children would be involved in the LPP and that their children would be questioned about their experiences and ideas. All the children in the settings were informed about the research project and the aim of providing the loose parts. They were free to ask questions to obtain more information, and it was ensured that the children understood that they could withdraw at any time [85]. None of the parents refused the participation of their child, and no child withdrew during the study. The names of individuals and settings were anonymized to maintain the privacy of the participants.

In line with previous research on and with children, we recognize that children’s expressions are influenced by their interactions with researchers and their assumptions [86]. Hence, in the data collection phase, as well as during the analysis, our understanding of children’s voices and their ability to convey them was inhibited, which shaped our interpretations of the data [16,87]. However, the researchers were responsive to the children’s world by assuring their autonomy and active participation and by displaying pedagogical sensitivity in their contact with the children [88,89,90].

One of the components of this study was to diversify children’s risky play, including allowing the children to expose themselves to potential peril. This approach raises ethical questions about adult responsibility [72]. To forestall dilemmas in the field, the professionalization program that was carried out before the loose parts intervention was started included the risk–benefit approach, which encourages practitioners to tolerate more risk in children’s play by assessing the developmental benefits [66,91,92]. The program, conducted in three sessions, focused on knowledge, attitude, and supervision of risky play. It also incorporated the facilitation and guidance of children’s risk-taking and loose parts play. In this way, the regular staff as well as the student researchers were aware of their non-intervening role, only intervening in children’s play in the case of serious possible physical harm that children could not predict. 

## 5. Results

The results of the analysis are presented in line with the four research questions, revealing three and subsequently four main themes (see Table 2). 

### 5.1. Children’s General Views on Risky Play

We found that the children could give many different examples of risky play in their lives. Our analysis focused on ranking the six categories of risky play that children most frequently pronounced. Foremost, they perceived risk to be merely related to physical risks. They most often connected their practices with the categories of height and speed. Rough-and-tumble play and play near dangerous elements, like water and fire, were also often detailed in children’s descriptions of risk in play, although less than height and speed. Dangerous tools and playing out of sight were the least mentioned.

*If I want to jump off my bunk bed, if I look down, it looks like it’s 10 m deep. It is scary, but then I just do it anyway.*

*(P.)*

Importantly, some children mentioned activities as risky play that are not defined in the six categories, like parkour, which refers to balancing and jumping from one feature to another. Furthermore, they talked about playing in the dark or during the night as risky.

*Hide and seek in the dark is exciting to do.*

*(S.)*

Children articulated their experiences that come with playing together, which frequently occurs in after-school childcare settings. They viewed such play as risky. They also shared some undesirable experiences, like when one child spoils play for another child. Children usually view these risks positively, as a natural part of playing together. They understand the need to figure out who dares and who does not during a certain play activity. They must indicate their own borders, and making agreements is necessary when rough-and-tumble play is going on.

*While we are playing rough, and I do not want things.*

*(A.)*

*You better go in there yourself; otherwise, we’ll push yóu over.*

*(B.)*

### 5.2. Children’s Play Experiences with Loose Parts

Both the interviews and diaries revealed a wide variety of play possibilities the children experienced. They expressed intense enthusiasm for the loose parts, which was sometimes in contrast with the dullness of the after-school childcare they were used to.

*This is stuff where we normally aren’t allowed to play with.*

*(K)*

*Now I do not have to be bored anymore outside.*

*(T.)*

*Usually I went inside to do my homework, what I don’t do anymore, ha ha.*

*(K.)*

Regarding their play with the loose parts, children frequently mentioned creative and risky play. They also said that before the intervention, they had had fewer opportunities for such play.

*We just went off the hill with the wheelchair and then went falling.*

*(S2.)*

*We always play astronaut; the parasol is then the satellite and the barrel is the rocket which rolls down the mountain. I then go after the barrel with the buggy or run with the parasol after it to have enough reach and then we call together.*

*(M.)*

The analysis also showed that children who did not play together before were now becoming playmates. They made plans together for what to do with the loose parts, which gave them enjoyment.

*She didn’t belong to the group, and now we are playing together.*

*(S2.)*

### 5.3. Children’s Actual Risky Play Experiences

In stating their practices with risk-taking in their play, the children were aware that their actions did not always lead to the most preferred outcome. They were fine with the consequences if it “went wrong”, and they seemed to deliberate on these implications before engaging in a risky activity.

*Sometimes it goes wrong, I got a bruise and a little bit of blood.*

*(K2.)*

*Because I am afraid that when I fall, I fall really hard. But I ignore that thought.*

*(L.)*

*It is okay if something goes wrong.*

*(B.)*

The children’s words show that they value risky play and that they experience fear and joy at the same time.

*It makes me happy, and it makes me less fearful.*

*(H.)*

*A bit scary, and yeah, it is fun!*

*(X.)*

The data indicated a strong desire to have opportunities to make their own decisions in risky play. With such opportunities, they feel free to push their boundaries and therefore dare to attempt something beyond their current skill level. By doing so, children feel more confident and trust their capabilities.

*That we think ourselves if it will succeed and have freedom to do this.*

*(G.)*

*It makes me happier and, that I dare more.*

*(P.)*

*Yes, then I am also proud of myself.*

*(G.)*

### 5.4. Children’s Opinion on the Role of Practitioners

According to the interviews and diaries, the children’s play plans were negatively affected by the actions of adults. There seemed to be many rules that hindered them from following their own interests and ideas in their play—for example, in rough-and-tumble activities.

*We are not allowed to play rough, and we just like it, I want wounds, ha ha.*

*(G.)*

*Now the loose parts are here, they (supervisors) don’t say that anymore (“play calmly!”).*

*(W.)*

*I just want them to leave us alone.*

*(G.)*

The children stated that they wanted to receive assistance according to their own criteria. They want to sort things out themselves in their risk-taking activities, and they are competent enough to ask for support if necessary.

*After a while they wanted to go faster. Then the supervisor made a ramp where they could roll off.*

*(observation F2o.)*

The children said they needed to be taken seriously and that they wanted to be involved in determining what kinds of boundaries were necessary to restrict their freedom in risky play. Children believed adult supervisors should express more confidence in children and take their opinions into account in a proper way.

*That we get more trust, we want to gain self-confidence, we just want so much more.*

*(G.)*

Lastly, adults should keep a suitable distance from the playing area where children experiment with risk-taking. Children understand that practitioners have a role in supervising and are present; however, adults need to be reluctant to say something or intervene in other ways.

*They must stay and watch, but that it is okay what we are doing.*

*(L.)*

In contrast to the children who were in favor of risky play, other children expressed no or little interest in risk-taking play.

*No, what I actually do a lot of is talk. I don’t really do activities. I am more into talking.*

*(K.)*

*Sometimes I have a little fear of heights. Then you just do something else.*

*(R.)*

*I prefer not to play when the risks are too great, and you can get hurt.*

*(W.)*

However, these comments are exceptions in the interviews and diaries. Most of the children engaged in risky play at their own level and according to their capabilities and interests—from carefully taking steps to being daring and reckless. These findings reveal the differences between children in their practices and understandings of risky play.

## 6. Discussion: Children’s Need for Risk and Challenge

In this study, children in seven after-school childcare settings were given opportunities to play with loose parts, which stimulated risky play. This play was facilitated through supportive guidance from the practitioners. Children’s perspective was the focus of the four distinct research questions on children’s views on risky play, their play experiences with loose parts, their actual experiences with risky play, and their opinion of adult supervisors. In this section, the findings are discussed, relating them to risky play, loose parts play theories, and SDT. We also discuss possible implications for supervising risky play practices in professional settings. 

### 6.1. Children’s Views on Risky Play: Physical and Social Risks as Part of Regular Play

The qualitative data of this study showed that the children’s opinions about their general experiences with risky play are mostly congruent with Sandseter’s [40] six categories of physical risks. Heights and speed were most frequently stated as risky, which might relate to the overrepresentation of those possibilities in children’s play environments, such as play structures, trees, bikes, and trikes. The mention of rough-and-tumble play as a favorite aligns with research showing that sensation-seeking children are attracted to physical, risky play that can cause injury [93]. Water is relatively omnipresent in the Dutch play environment, which might explain children’s statements that playing near and with water is exciting and risky. 

This study revealed some new discoveries about existing risky play categories. Firstly, an interesting result was that children stated play situations that do not fit neatly into the categories and subcategories of risky play [23]. It was found that parkour (i.e., leaping from one outdoor place to another) was not present in the original categories; however, this has been mentioned in past studies as an element of risky play [85]. Furthermore, we found that the children viewed playing in the darkness, in darkened spaces, or after twilight as an important and challenging play activity. This is relatively unrecognized in children’s risky play research. For example, Prešlenkova [94] claims that there is a lack of study on the benefits of free play in the dark. Finally, we found that the children considered playing together to be risky, as it has the chance of going wrong. This aligns with the proposed re-conceptualization from Cooke et al. [25], which extends risky play from pure physical risk to social and emotional risk during play. Children in the present study had a positive perception of the risks perceived from playing together; they perceived challenging each other and some mild peer pressure as part of normal play. In this way, children are positioned to make agreements, and they learn to state their own borders.

### 6.2. Children’s Play Experiences with Loose Parts: Change and Novelty

Like Bundy et al. [62], we expected that the introduction of loose parts in after-school childcare settings would alter children’s play. The outcomes from interviews and observations clearly indicate that children’s opportunities to play changed positively; their play experiences broadened and led to new play arrangements with other children. From an SDT perspective, there were changes in relatedness and autonomy that contributed to children’s intrinsic motivation to engage in play with loose parts.

Firstly, the introduction of loose parts pushed children more outdoors, giving new insight into adults’ effortless labeling of children as “indoor children” [95]. When the play space and materials offer adequate affordances [57] for every child, like complexity, versatility, and flexibility in loose parts, it is more likely that all children will be attracted to the outdoors as it is less tedious. We conclude that this is even more important for older children who drop out of after-school childcare because they find the setting boring [96]. Secondly, the children reflected through this project on how they played with loose parts, resulting in descriptions of original and novel play situations where imagination and inventiveness increased. Moreover, we found that children described all kinds of forms of risky play that were provided by the loose parts. As one of the intentions of this study was to find out if loose parts would facilitate risk-taking play, the data validate this assumption. Future research should focus on which kinds of loose parts are especially appropriate for children’s levels of risk-taking. Thirdly, in this study, the children said that the loose parts prompted them to play with different children than before. This trend might originate from children having an interest in the same loose part and making new connections because of that shared interest. This new connection leads children to make plans together, to communicate, and to experience increased pleasure. Overall, we found that the loose parts intervention confirmed earlier research based on professional observations, such as the study of Hyndman et al. [64]. Likewise, in another study, children’s play was described by the principal as “*busy, motivated and engaged“* [97], and increasing social development and cooperation were noted.

### 6.3. Children’s Actual Risky Play Experiences: Positivity and Trust

In this study, three distinct themes were identified to reflect how children viewed risky play. Firstly, we found that the children instinctively recognized the chances of hurting themselves by engaging in risky play and that they accepted the possible consequences. This finding aligns with prior research that suggests that children can provide valid self-reports of their willingness to take risks and that children are aware of their risk-taking in play and can report on these actions [98]. Secondly, we conclude that children associate positive emotions with risky play activities. Sandseter [72] termed the ambiguous feelings of joy and fear that come with risky play as “*scary-funny*” (p. 100), since individual children in her research described their dual experiences with this phrasing. Misinterpretation of children’s fear, which they see as a natural part of their play, can lead practitioners to habitually intervene because they want to protect children against undesirable emotions, thus constraining children’s opportunities to discover their boundaries. This restraint would be an unwanted outcome, as the third theme showed that children want to make their own decisions in risky play, which leads them to trust their own actions. In this way, children can expand their risk competence, which is shown to be strengthened by facilitating the possibilities of risky play activities [39].

Thus, giving children the opportunity to take risks on their own terms gives them a sense of self-confidence and mastery, connecting to two of the three universal needs of SDT: autonomy and competence. Moreover, children who take risks in play learn to trust themselves, understand their capabilities, recognize limits, and have knowledge of when to ask for assistance [12]. The third psychological need, relatedness, appears in the final themes.

### 6.4. Children’s Opinion on the Role of Practitioners: Child-Led Collaboration

This study found a strong judgment of children about the attitude of practitioners and how the possibilities to engage in risky play are affected by a non-permissive and intervening adult. We conclude that children see adults as interfering with their freedom to play in their own way. They understand that in an after-school setting, practitioners have to be present and are responsible; however, children want them as distant as possible so that practitioners do not intrude in their risk-taking play. We found that any necessary general restrictions or rules governing risky play should be made in consultation with children, who want to express their vision. That vision should be taken seriously if children’s autonomy is to be promoted. Children were clear that they have no need for adult suggestions or advice during risky play activities. Help is acceptable, but only when children ask for it. Based on SDT, the social nature of activities during risky play involves making choices, relating to other people (children and adults), and developing skills that help them take some control of their lives [35]. Meeting children’s innate need for SDT’s category of relatedness (including in risky play) may be a sensitive task for practitioners because they have to shift from distance to involvement. Van Manen articulates this as pedagogical sensitivity, *“which pertains to doing the right thing for this child in this situation. It is about tactful action as ‘an immediate involvement in situations where I must instantaneously respond, as a whole person, to unexpected and unpredictable situations’”* [99].

This sensitivity can be connected to six “interactional skills” used in Dutch after-school childcare settings: sensitive responsiveness, respect for autonomy, structuring and setting limits, talking and explaining, stimulating development, and guidance of interactions between children [100]. These six skills can all support children’s risky play [101]. Thus, we conclude that practitioners in after-school childcare have a duty of care to reinforce risky play practices in a sensitive and receptive manner.

### 6.5. Strengths and Limitations of This Study

Several strengths and limitations may have impacted the results of this study. One strength of this study is its qualitative approach. By eliciting individual children’s understanding of risky play, this study enriches the discourse on how to connect to children’s needs in their risk-taking play. This study complements the recent literature by exploring children’s perspectives on risky play, including those perspectives in relation to supervising adults [22]. The view of children can help practitioners implement interventions that facilitate risky play. 

Of course, one limitation of the present study is that different childcare settings and child populations were included; however, the findings were not specified or identified by age or background. Future research should explore the possible differences and similarities between these aspects. Moreover, this study was only a six-week intervention, so the long-term effects on children’s judgment and perceptions of increased risky play possibilities were not considered. Although enduring positive experiences could be predicted, future research is needed on this specific approach. Another limitation is that giving the responsibility for the collection of the data to the student researchers had certain constraints and difficulties. The first author purposefully adopted a facilitating role to position students as partners in the research for wide-ranging learning possibilities [102]. Improvements could have been made in implementing data collection methods and monitoring accurate registration.

## 7. Conclusions: Risk as an Inherent Aspect of Children’s Play

The deliberate creation of uncertainty is present in much playing, and therefore most play situations can be considered risky in some way. Children know this and accept it. However, there is considerable variation in the way risk is perceived, resulting in different child and adult appraisals of risky play. By examining children’s perceptions of risky play in after-school childcare settings in this study, we gained an in-depth understanding of their experiences, their risky play with other children, and their relationship with their professional caretakers during risky play. The loose parts intervention was helpful for tapping into riskier play practices, which children could accurately express in words. 

Our first aim was to contribute to the scientific knowledge on the concept of risky play. By endorsing parkour as a subcategory and proposing playing in the dark as a new subcategory, the six risky play categories can be more closely described in terms of what children express. Furthermore, we made the case that in children’s perceptions of risky play, they need autonomy, competence, and relatedness, as theorized in SDT. The need for relatedness includes the relationship of children with adults, with a more trusting and relaxed atmosphere regarding risk-taking. Moreover, it also includes relationships among children through social interaction and learning processes. In the context of such relationships, children make new friends and experience fun when they engage in risky play. 

Another goal of this study was to encourage practitioners to reconsider their approach to supervising risky play. Children can articulate their perceptions of the attitudes of the adults supervising them and can give clear advice on how to not act regarding risky play. Listening to children can make professional caretakers more aware of the fact that children are competent appraisers and assessors of risk. It is important for children to make their own choices as much as possible in their risky play to experience a sense of freedom. The risky possibilities of the play space, the availability of resources like loose parts, participation by other children, and the proximity of adults influence children’s choices pertaining to risky play. The proposed pedagogical sensitivity could be a professional tool for exercising appropriate distance from children. In this way, the outcomes of this study can contribute to changing practices in after-school childcare from the perspectives of protection and proximity. Specifically, instead of quickly intervening in children’s risky play, caregivers can move toward a more relaxed, wait-and-see attitude. Realizing that children have an innate need for autonomy and that they can make their own risk assessments in their play, practitioners can trust them, knowing that this trust fosters self-regulation and resilience and is thus essential for healthy maturation. 

## Figures and Tables

**Table 1:** Context, age, and number of children in participating childcare settings.

Context	Age of Children with Access to LPP	Number of Children *
Outdoors (“forest school”)	4–12	60
Sports focus	4–6	20
	7–12	30
Farm site	4–12	90
Scouting accommodation	4–8	30
Urban low socioeconomic status (SES)	4–7	120
	6–12	40
High SES village	4–12	40
Regular urban context	9–12	45

* Dutch after-school childcare is organized by groups of 20 children. Note, this number is not specified in gender, because it was not planned which specific children would participate in the LPP, as the presence of children varied from day to day. Children were not present every day of the week, and the LPP days were decided by the team.

**Table 2:** Summary analysis: themes and descriptions.

Research Questions	Themes	Description
Children’s general views on risky play	“Great heights,” “speed,” “rough & tumble”, and “dangerous elements”	Most occurring categories of risky play
	“Parkour” and “dark/night”	Supplemental, as these are not defined in the six categories
	Positive about “playing together” risks	Making agreements and stating own borders
Children’s play experiences with loose parts	Change in play	More outdoors, more opportunities
	Creative and risky play	Novelty in play
	Now playing along with others	New playmates, making plans, and having fun
Children’s actual risky play experiences	Chance of hurting	Acceptance of possible consequences
	Different emotions	Expressed positively
	Doing it themselves	Having trust, and daring
Children’s opinion on the role of practitioners	Adults are a disturbing factor	A non-permissive environment
	Help is acceptable	Only when children ask for it
	Frameworks/borders in consultation	Children have their own vision
	Keep appropriate distance	Present but not intrusive

## Data Availability

The data presented in this study are available upon request from the corresponding author.
